# 

**Published:** 1883-10-20

**Authors:** 


### TABLE OF CONTENTS.

ORIGINAL AND SELECTED ARTICLES.

Obstetric Society of Philadelphia, 361. Some General and Special Facts Concerning Syphilis, 365. Baltimore Medical Association, 368. Therapeutic Action of Gelsemium, by E. W. Lane, M.D., Scarboro, Ga., 371. A Doctor's Diploma Before a Jury, 373. Syphilis from Accidental Causes, 374. Arnica Poisoning, by Wm. A. Thom, Jr., M.D., Norfolk, Va., 375. Curious Accident at the Paris Electrical Exhibition, 376.

ABSTRACTS AND GLEANINGS.

Country and City Doctors, 377. Strychnine, 379. Blood-Letting, 380. Neuralgia, 381. The Physiological Effect of Coffee; Effect of Alum Gargle upon the Teeth, 382. Cannabis Indica; A Valuable Remedy in Hemorrhagia, 383. Paraldehyde and Acetal as Hypnotics. 384. Treatment of Tonsillitis; Treatment of Sporadic Cholera, 385. Cellulose as a Dressing; Commotio Retinæ, or Some of the Effects of Direct and Indirect Blows to the Eye, 386. New Reasons for Woman's Nursing; Action of a Mixture of Vapor of Chloroform and Air, 387. Iodine Painting in Small-pox, Arbor-Vitæ; Urethral Stricture, 388. Pulse and Temperature in Typhoid Fever; Calomel in Diptheria; Good Sight; Acute Tonsillitis, 389. Enlarged Tonsils; Cholera Infantum; The Permanency of Eserine Solutions; Menorrhagia—Cannabis Indica; Effect of Pilocarpin upon Color of the Hair, 390.

SCIENTIFIC ITEMS.

Limits of Germination; First Idea of the Telephone; Shortness of Time in Dreams, 391. Blood Poison, Spiders and the Electric Telegraph, 392.

PRACTICAL NOTES AND FORMULÆ.

Eczema of the Face; Acute Rheumatism; Thompson's Eye Water. 393. Squibb's Cholera Remedy; Compound Parsley Mixture; Syrup of Coffee; Creasote Wine, 394. S. S. S., To Allay the Itching in Eczema; Remedy for Earache; Hydrated Oxide of Iron; Whooping Cough Specific, 395. Flatulent Dyspepsia; Intense Itching; Sir Benjamin Brodie's Prescription for Gout; Tetter of the hands; Resorcin in Cholera Infantum; To Prevent Pitting in Smallpox; Urticaria, 396.

EDITORIALS AND MISCELLANEOUS.

Editorial Notices; Transactions of the Medical Association of Georgia, 34th Annual Session, 397. Southern Medical College, Atlanta; Pharmacy in America. Deaths of Physicians· Pamphlets Received, 399; Receipted; Special Notices, 400.

